# Cerebellar contributions to visuomotor adaptation and motor sequence learning: an ALE meta-analysis

**DOI:** 10.3389/fnhum.2013.00027

**Published:** 2013-02-07

**Authors:** Jessica A. Bernard, Rachael D. Seidler

**Affiliations:** ^1^Department of Neurology, University of Colorado Denver School of MedicineAurora, CO, USA; ^2^Department of Psychology, University of MichiganAnn Arbor, MI, USA; ^3^School of Kinesiology, University of MichiganAnn Arbor, MI, USA; ^4^Neuroscience Graduate Program, University of MichiganAnn Arbor, MI, USA

**Keywords:** cerebellum, sequence learning, visuomotor adaptation, working memory, meta-analysis

## Abstract

Cerebellar contributions to motor learning are well-documented. For example, under some conditions, patients with cerebellar damage are impaired at visuomotor adaptation and at acquiring new action sequences. Moreover, cerebellar activation has been observed in functional MRI (fMRI) investigations of various motor learning tasks. The early phases of motor learning are cognitively demanding, relying on processes such as working memory, which have been linked to the cerebellum as well. Here, we investigated cerebellar contributions to motor learning using activation likelihood estimation (ALE) meta-analysis. This allowed us to determine, across studies and tasks, whether or not the location of cerebellar activation is constant across differing motor learning tasks, and whether or not cerebellar activation in early learning overlaps with that observed for working memory. We found that different regions of the anterior cerebellum are engaged for implicit and explicit sequence learning and visuomotor adaptation, providing additional evidence for the modularity of cerebellar function. Furthermore, we found that lobule VI of the cerebellum, which has been implicated in working memory, is activated during the early stages of explicit motor sequence learning. This provides evidence for a potential role for the cerebellum in the cognitive processing associated with motor learning. However, though lobule VI was activated across both early explicit sequence learning and working memory studies, there was no spatial overlap between these two regions. Together, our results support the idea of modularity in the formation of internal representations of new motor tasks in the cerebellum, and highlight the cognitive processing relied upon during the early phases of motor skill learning.

## Introduction

**Figure 1:**
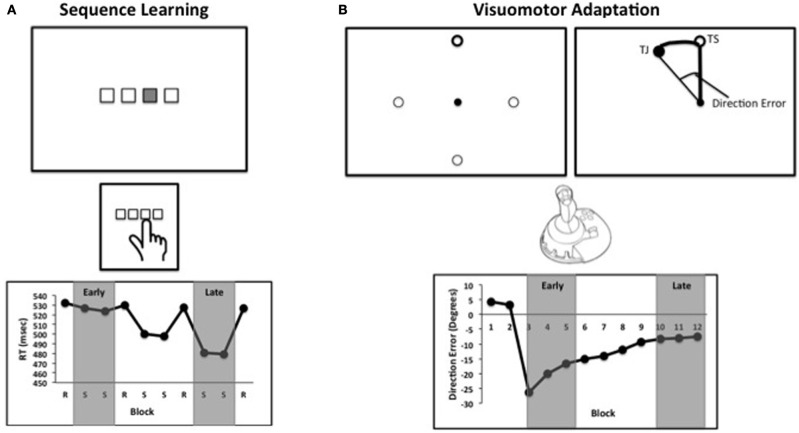
**(A)** Schematic of a standard sequence learning task. Stimuli corresponding to buttons on a response box or keyboard are presented on a computer screen. The sequence is presented by highlighting a location, and the participant presents the corresponding button. Blocks alternate between sequence (S) presentations, and the presentation of locations in random order (R). **(B)** A schematic of a visuomotor adaptation task. Participants are presented with one of four targets on a computer screen, and are asked to move the cursor to the highlighted circle (top left). After several practice blocks, the feedback is rotated with respect to the participant's movement. Participants attempt to move toward the target in screen coordinates (TS), but due to the rotation subjects move toward the closed circle (TJ, target location in joystick coordinates), which is not visible to participants (top right). Direction error refers to the angle between the line from the center to the target and the line from the central to the location of the joystick at the time of peak velocity. This example is similar to what would be seen during early learning. In both panels, example data are presented. In the studies included in our meta-analysis, early and late learning were defined by the experimenters. Examples of the early and late learning phases for each task are highlighted in gray.

Individuals are able to learn to use new tools and can turn novel movements into accomplished skills through practice. This process recruits a diverse network of cortical and subcortical brain regions (Jenkins et al., 1994; Imamizu et al., 2000; Doyon et al., 2002; Lehéricy et al., 2005; Seidler et al., 2006), though the neural substrates vary somewhat based on task type (c.f. Rauch et al., 1995; Honda et al., 1998; Schendan et al., 2003). Several different paradigms have been used to investigate motor skill learning. These commonly include visuomotor adaptation and motor sequence learning. Visuomotor adaptation requires individuals to adapt movements to distorted visual feedback (e.g., Imamizu et al., 2000; Seidler et al., 2006). The sensory information provided to the participant does not match the movement they have made, and as such the participant needs to modify their movement to produce the appropriate result. Motor sequence learning requires individuals to learn novel patterns of movements, often made with the fingers (Figure 1). Based on cues provided to the individual, a new movement sequence is practiced and learned. Within the domain of motor sequence learning both implicit and explicit paradigms are used (e.g., Schendan et al., 2003). During implicit sequence learning, the goal of learning a new sequence is unknown to the participants, and the sequence is often embedded within other movements. Conversely, during explicit sequence learning, the goal of learning the sequence is made clear at the outset of the task. One brain region that has been consistently implicated in motor learning is the cerebellum. Cerebellar activation has been observed in a variety of motor learning tasks including visuomotor adaptation (Imamizu et al., 2000, 2003; Anguera et al., 2010) and both implicit and explicit motor sequence learning (Jenkins et al., 1994; Grafton et al., 2001; Lehéricy et al., 2005; Orban et al., 2010). Cerebellar circuits have also been implicated in associative learning paradigms such as eye-blink conditioning (Woodruff-Pak et al., 2000, 2001; Cheng et al., 2008).

In particular, the cerebellum is thought to play a role in the formation of internal representations of actions that allow for the smooth execution of motor skills (Ramnani, 2006; Ito, 2008). Learning and formation of these representations is thought to rely on error signals based on feedback from prior performance (Ito, 2000). The result of this learning is a new internal model of a particular task. Indeed, the engagement of the cerebellum during the learning of a new motor task changes as the course of learning progresses (Imamizu et al., 2000). During a locomotor adaptation task, cerebellar excitability is decreased over the course of the task as measured by the degree of cerebellar brain inhibition of the motor cortex (Jayaram et al., 2011). Furthermore, the degree to which cerebellar brain inhibition decreased was strongly associated with learning of the locomotor adaptation task such that those with the greatest decreases in cerebellar excitability learned best. This decrease in excitability was suggested to be related to synaptic long-term depression (Jayaram et al., 2011). Also using non-invasive brain stimulation it has been demonstrated that the cerebellum is associated with the learning of a visuomotor adaptation task, while the primary motor cortex is associated with retention of learning (Galea et al., 2011). Relatedly, different neural substrates are engaged during performance of a task shortly after learning, including the cerebellum (Shadmehr and Holcomb, 1997). This is indicative of changes in and consolidation of the internal model of a particular action.

With that in mind, one is likely to learn multiple motor skills. The question then becomes whether or not the cerebellum then forms distinct internal models for these different motor skills. It has previously been suggested that multiple internal models are present in the cerebellum. This has been conceptualized in the computational model know as MOdular Selection And Identification Controller (MOSAIC; Wolpert and Ghahramani, 2000; Imamizu et al., 2003). Imamizu et al. (2003) tested this idea by having individuals learn to use a computer mouse under two novel visuomotor mappings. The visual feedback of the mouse was rotated, and in a separate condition, the velocity of the feedback was also manipulated. Over the course of learning in these two conditions, they found distinct regions of cerebellar activity, supporting modular internal models in the cerebellum (Imamizu et al., 2003). Imamizu and colleagues (2003) noted that this work serves as an extension of the MOSAIC theory in that the regions of cerebellar engagement associated with the internal models of these two conditions are in lateral regions of the cerebellum more associated with cognitive functions. Regardless, the MOSAIC theory can be further tested in the motor domain through the use of meta-analysis, as there are now numerous studies of motor learning across a variety of motor task domains. However, given the potential storage capacity issues with strictly modular representations of internal models, there may be overlapping cerebellar regions associated with motor tasks that require similar types of cognitive processing for learning, or are similar in task domain (for example, implicit and explicit sequence learning).

In addition to investigating the MOSAIC theory through the use of meta-analysis, this method also allows us to investigate the potential cognitive contributions of the cerebellum to motor learning. Compared to the more automatic performance that occurs in late learning, the early stage of learning is thought to be cognitively demanding (Fitts and Posner, 1967). Indeed, the rate of early learning during a visuomotor adaptation task has been correlated with individual differences in spatial working memory ability, as measured using the card rotation task (Anguera et al., 2010). Furthermore, this work demonstrated that in this early learning phase, there is engagement of prefrontal and parietal brain regions that are also associated with the performance of a working memory task involving mental rotation (Anguera et al., 2010). Relatedly, visuospatial and verbal working memory have also been implicated in motor sequence learning. Visuospatial working memory capacity is correlated with explicit motor sequence learning and the formation of motor chunks (Bo and Seidler, 2009; Bo et al., 2009). In implicit sequence learning paradigms, both visuospatial and verbal working memory are correlated with improved performance (Bo et al., 2011, 2012). Additionally, individuals with high working memory capacity learn sequences better when executive attention is required relative to those with low working memory capacity (Unsworth and Engle, 2005).

The posterior and lateral regions of the cerebellum have been associated with the performance of working memory tasks (Chen and Desmond, 2005a,b; Kirschen et al., 2005, 2010; Stoodley and Schmahmann, 2009; Stoodley et al., 2010, 2012). While these regions have been investigated using working memory paradigms, it remains unknown whether the same sites are also engaged during the learning of new motor skills. Given that prefrontal and parietal regions associated with working memory are also engaged during early visuomotor adaptation learning (Anguera et al., 2010), the same may be true for the cerebellum. Though more lateral regions of the cerebellum have been recently implicated in complex motor tasks (Schlerf et al., 2010), perhaps due to the cognitive demands of those tasks, there have been no investigations of whether the same cerebellar regions are engaged for both working memory and motor skill learning. Again, meta-analysis allows for assessment of this question.

Here our goal was to investigate the cerebellar contributions to both sensorimotor adaptation and sequence learning. Cerebellar activation has been seen in implicit and explicit sequence learning and visuomotor adaptation, along with both spatial and verbal working memory (Hazeltine et al., 1997; Thomas et al., 1999; Daselaar et al., 2003; Haaland et al., 2004; Krakauer et al., 2004; Chen and Desmond, 2005a,b; Lehéricy et al., 2005; Seidler et al., 2006; Anguera et al., 2007; Schendan and Stern, 2007; Stoodley et al., 2010). Given that working memory capacity is correlated with these three types of learning (Bo and Seidler, 2009; Bo et al., 2009, 2011, 2012; Anguera et al., 2010, 2011), it may be the case that a single cerebellar modular region underlies all three types of learning. Though the cerebellum and basal ganglia show dissociated activity in the later stages of learning, both are active in the earlier stages of learning for both sequence learning and visuomotor adaptation (Doyon and Benali, 2005). One possibility is that the overlapping neural substrates of learning in the cerebellum may be due to the involvement of the cerebellum in working memory processes, particularly given that working memory is important for both sequence learning and visuomotor adaptation (Bo and Seidler, 2009; Bo et al., 2009, 2011, 2012; Anguera et al., 2010, 2011). However, because cerebellar engagement changes over the time course of learning (Imamizu et al., 2000, 2003), it may be oversimplified to look at just general overlap across these task types. Thus, we will investigate overlap in cerebellar activation across studies for working memory tasks with that of explicit sequence learning, implicit sequence learning, and visuomotor adaptation, taking into account the stages of learning (early vs. late) whenever possible. This approach will help to refine our view of cerebellar functions and modularity for cognitive and motor behaviors. In particular, investigations of the early and late stages of learning will provide further insight into the formation of internal models and allow for an additional test of the MOSAIC theory (Wolpert and Ghahramani, 2000; Imamizu et al., 2003) in the motor domain.

We used activation likelihood estimation (ALE) meta-analysis (Turkeltaub et al., 2002; Laird et al., 2005; Eickhoff et al., 2009), implemented using the GingerALE software package, to investigate the cerebellar regions involved in both motor sequence learning and visuomotor adaptation as well as working memory. Given the number of task domains, and the time necessary to assess learning, it would be extremely challenging to investigate all of these tasks in one functional neuroimaging study in order to answer the questions at hand. Meta-analysis, however, provides a reasonable solution. ALE meta-analysis pools coordinates in standard space across studies, and treats them as spatial probability distributions. Overlap among these regions is assessed through permutation testing, and the result is an ALE statistic for regions across studies with significant overlap (thresholded and corrected for multiple comparisons; Turkeltaub et al., 2002; Laird et al., 2005; Eickhoff et al., 2009). Studies demonstrating cerebellar activation during visuomotor adaptation, explicit and implicit sequence learning, as well as both spatial and verbal working memory tasks were combined in this meta-analysis. We hypothesized that visuomotor adaptation and motor sequence learning would engage similar motor regions of the cerebellum during early learning, but additional distinct regions associated with spatial and verbal working memory processes, respectively, would be engaged as well. We further hypothesized that distinct regions of the cerebellum would be involved in the later stages of learning and the formation of internal models, consistent with the MOSAIC theory (Wolpert and Ghahramani, 2000; Imamizu et al., 2003) which suggests a modular organization of representations in the cerebellum.

## Methods

### Literature review

Papers were identified through three PubMed (http://www.ncbi.nlm.nih.gov/pubmed/) searches. Searches for papers investigating visuomotor adaptation, motor sequence learning, and working memory were conducted separately using the following search terms: “sensorimotor adaptation AND imaging,” “motor sequence learning AND imaging,” and “working memory AND imaging.” Additionally, the searches used the limits “Humans,” “English,” and “Adult 19-44 years.” These searches resulted in 45, 149, and 1997 papers, respectively. We also consulted a recent review of motor learning and included related work on sensorimotor adaptation not found in our PubMed search (Seidler, 2010). We followed the same exclusion criteria as reported by Stoodley and Schmahmann (2009). That is, we excluded papers that did not use functional imaging techniques, did not report any coordinates in the cerebellum, did not report coordinates in either Montreal Neurological Institute (MNI; Collins et al., 1998) or Talairach (Talairach and Tournoux, 1988) space, investigations with incomplete coverage of the cerebellum, those using only region of interest analyses, and clinical or aging studies that did not report a healthy young adult control group. Additionally, we excluded studies where the learning of the adaptation task or sequence was completed outside of the scanner (that is, the early learning phase was not scanned), and those that did not have subjects overtly perform the task (e.g., studies that investigated mental rehearsal of a sequence and the resultant learning outcomes), along with studies that did not use standard contrast analyses (for example, those using independent components analysis). Studies of working memory were limited to the spatial and verbal domains, consistent with tasks found to be associated with motor learning (Bo and Seidler, 2009; Bo et al., 2009, 2011, 2012; Anguera et al., 2010, 2011). Thus, we excluded studies with emotional, auditory, and visual manipulations. After excluding studies that did not meet our criteria, 5 studies of visuomotor adaptation, 18 studies of sequence learning, and 44 studies of working memory remained (9 of spatial working memory, and 35 of verbal working memory). Finally, for our analyses of sequence learning, we divided our studies into those investigating implicit sequence learning (7 studies) and those investigating explicit sequence learning (the remaining 11 studies). Studies of explicit sequence learning were further divided, grouping those investigating early and late learning separately (5 studies in each category). The number of studies included in each of our task domains is consistent with the number of studies used in similar recent ALE meta-analyses of cerebellar function (Stoodley and Schmahmann, 2009; E et al., in press). These investigations included between 2 studies (somatosensory task domain; Stoodley and Schmahmann, 2009) and 26 studies (working memory domain; E et al., in press). Though our initial goal was to compare early and late learning across all three motor tasks, none of the studies meeting our criteria for both visuomotor adaptation and implicit sequence learning included analyses based on learning stage, and we were therefore unable to complete the analysis of learning stage on these two task domains. For explicit motor sequence learning, early and late learning were typically defined within a single practice session. The first half of learning was compared to the second half of learning. However, in one instance (van der Graaf et al., 2004) learning was compared across two sessions with practice occurring for several days in between the two sessions. Table 1 presents the studies included in our analyses, along with the sample size, imaging modality, the number of cerebellar foci, and a brief description of the tasks and contrasts resulting in those foci for each study.

**Table 1:** **Studies included in the meta-analysis, organized by category**.

**Study**	**Imaging modality**	***N***	**Task**	**No. of foci**
**VISUOMOTOR ADAPTATION**				
Luauté et al. (2009)	1.5 T fMRI	11	Prism adaptation	1
Anguera et al. (2007)	3 T fMRI	16	Adaptation to perturbed visual feedback using a joystick, conjunction of early and late learning	2
Seidler et al. (2006)	3 T fMRI	26	Adaptation to perturbed visual feedback using a joystick	1
Graydon et al. (2005)	4 T fMRI	12	Adaptation to perturbed visual feedback using a joystick	1
Krakauer et al. (2004)	PET	12	Moving target to cursor under rotated or varied gain feedback	2
**SEQUENCE LEARNING**				
Rose et al. (2011)	3 T fMRI	15	Implicit sequence learning, relative to random blocks, increased activation over course of learning	1
Rieckmann et al. (2010)	1.5 T fMRI	14	Implicit serial reaction time task (SRTT), increased activity in the second vs. the first half of learning paradigm	2
Albouy et al. (2008)	3 T fMRI	90	Implicit occulomotor sequence learning, activation increases and decreases associated with improvement, and learning main effects over time	4
Bischoff-Grethe et al. (2004)	1.5 T fMRI	24	Implicit sequence learning, with incompatible stimulus-response mapping, activation decreases across learning	4
Daselaar et al. (2003)	1.5 T fMRI	26	Implicit sequence learning, relative to random button presses	1
Grafton et al. (2002)	PET	8	Implicit SRTT (using left hand) with background tone counting task, activation decreases across learning	1
Hazeltine et al. (1997)	PET	11	Implicit SRTT with and without background tone counting task, activation decreases across learning	3
Lin et al. (2011)	3 T fMRI	16	SRTT with explicit awareness, comparing repetitive and interleaved practice	2
Orban et al. (2010)	3 T fMRI	16	Explicit sequence learning, areas modulated relative to increased performance, and main effect of learning relative to a tapping control	5
Bapi et al. (2006)	4 T fMRI	6	Explicit sequence learning under visual and motor rotation, activation relative to control in early and late learning	4
Floyer-Lea and Matthews (2005)	3 T fMRI	15	Explicit sequence learning using force changes, increases and decreases during early relative to later learning	2
Lehéricy et al. (2005)	3 T fMRI	14	Explicit sequence learning, main effects relative to control sequence, and activation decreases related to learning after practice outside scanner	4
Heun et al. (2004)	1.5 T fMRI	10	Explicit sequence learning and retrieval compared to random finger tapping	4
van der Graaf et al. (2004)	1.5 T fMRI	12	Double serial reaction time (DoSRT) task, two scan sessions with half of the subjects practicing in between, relative to a visual control, compared across scan sessions	8
Müller et al. (2004)	1.5 T fMRI	8	Explicit sequence learning, main effects of learning relative to tapping control in early and late phases	3
Haaland et al. (2004)	1.5 T fMRI	14	Explicit sequence learning of varying complexity, right hand greater than left hand performance activation, and complex greater than simple sequences	4
Müller et al. (2002)	1.5 T fMRI	7	Explicit sequence learning relative to tapping control task in the early and late phases of learning	4
Sakai et al. (2002)	PET	8	Explicit sequence learning, learning related increases in activation relative to random ordered control	1
**SPATIAL WORKING MEMORY**				
Blokland et al. (2011)	4 T fMRI	319	Spatial n-back task, 2-back relative to 0-back	5
Roebling et al. (2009)	1.5 T fMRI	20	Memory for location of shapes in a 5 × 5 grid, compared to a shape identification task	2
Cerasa et al. (2008)	1.5 T fMRI	30	Spatial n-back task, 2-back relative to 0-back	2
Leung et al. (2007)	3 T fMRI	14	Memory for location in a 4 × 4 grid with spatial updating relative to location comparison	3
Schendan and Stern (2007)	3 T fMRI	20	Mental rotation compared to object discrimination control task	3
Schöning et al. (2007)	3 T fMRI	30	Mental rotation of 3D objects relative to looking at 3D objects	13
Bor et al. (2001)	PET	10	Spatial span relative to pointing to illuminated locations	1
Thomas et al. (1999)	1.5 T fMRI	6	Spatial n-back task compared to button pressing control task	1
**VERBAL WORKING MEMORY**				
Joseph et al. (2012)	1.5 T fMRI	10	Verbal n-back task, 2-back relative to 0-back	5
Stoodley et al. (2012)	3 T fMRI	9	Verbal n-back task relative to responding to the presentation of the letter “X”	3
Schulze et al. (2011)	3 T fMRI	16	Modified Sternberg working memory task presenting tonal syllables, relative to the presentation of pink noise	5
Stoodley et al. (2010)	3 T fMRI	1	Verbal n-back task relative to responding to the presentation of the letter “X”	4
Michels et al. (2010)	3 T fMRI	16	Sternberg working memory task with 5 letters relative to 2 letters	6
Gruber et al. (2010)	1.5 T fMRI	18	Sternberg working memory during articulatory and non-articulatory rehearsal relative to letter-case judgments	4
Schneider-Garces et al. (2010)	3 T fMRI	17	Sternberg working memory task, increasing activation associated with increased load	1
Kirschen et al. (2010)	3 T fMRI	16	Sternberg working memory task, comparing high relative to low load across aural and visual stimulus presentation	16
Roebling et al. (2009)	1.5 T fMRI	20	Sternberg working memory task relative to letter-case judgments	2
O'Hare et al. (2008)	3 T fMRI	8	Sternberg working memory task investigating load-dependent activation	4
Koppelstaetter et al. (2008)	1.5 T fMRI	16	Verbal n-back task, 2-back relative to 0-back	1
Scheuerecker et al. (2008)	1.5 T fMRI	23	Verbal n-back task, 2-back relative to 0-back	1
Hayter et al. (2007)	3 T fMRI	15	Paced Auditory Serial Addition Test (PASAT), adding relative to repeating letters	4
Walter et al. (2007)	1.5 T fMRI	17	Sternberg working memory task at three loads relative to responding to the presentation of the letter “X”	6
Chang et al. (2007)	1.5 T fMRI	14	Sternberg working memory task, load-dependent activation	6
Caseras et al. (2006)	1.5 T fMRI	12	Verbal n-back task, linear increase in activation as a function of load	1
Knops et al. (2006)	1.5 T fMRI	16	Verbal n-back task, 2-back relative to 1-back	2
Mu et al. (2005a)	3 T fMRI	33	Sternberg working memory task relative to viewing an asterisk array	1
Mu et al. (2005b)	3 T fMRI	33	Sternberg working memory task with 3 and 6 letters relative to viewing an asterisk array	2
Wolf and Walter (2005)	1.5 T fMRI	15	Sternberg working memory task compared to responding to the presentation of the letter “X,” and load-dependent effects	3
Chen and Desmond (2005a)	3 T fMRI	17	Sternberg working memory task relative to a motor rehearsal control task	1
Chen and Desmond (2005b)	3 T fMRI	15	Sternberg working memory task, load-dependent activations	9
Audoin et al. (2005a)	1.5 T fMRI	18	Paced Auditory Serial Addition Test (PASAT), adding relative to repeating letters	1
Audoin et al. (2005b)	1.5 T fMRI	10	Paced Auditory Serial Addition Test (PASAT), adding relative to repeating letters	1
Kirschen et al. (2005)	3 T fMRI	16	Sternberg working memory task, load-dependent activations	5
Tomasi et al. (2005)	4 T fMRI	30	Verbal n-back task relative to the presentation of nonsense characters	3
Meyer-Lindenberg et al. (2005)	PET	24	Verbal n-back task, 2-back relative to 1-back	2
Mendrek et al. (2004)	1.5 T fMRI	8	Verbal n-back task, 2-back relative to 1-back	2
Cairo et al. (2004)	1.5 T fMRI	18	Sternberg working memory task, load-dependent activation	5
Crottaz-Herbette et al. (2004)	1.5 T fMRI	14	Verbal n-back task, s-back relative to button press when the number 3 was presented	1
Veltman et al. (2003)	1.5 T fMRI	21	Sternberg and verbal n-back tasks, load related increases in activation	2
Kim et al. (2003)	PET	12	Verbal n-back task, 2-back relative to button press control when a circle is presented	1
Desmond et al. (2003)	3 T fMRI	13	Sternberg working memory task, high relative to low load	5
Henson et al. (2000)	2 T fMRI	6	Sternberg working memory task relative to a letter matching control	3
Honey et al. (2000)	1.5 T fMRI	22	Verbal n-back task relative to responding to the presentation of the letter “X”	1

The sequence learning tasks required subjects to learn novel sequences of movements, typically through finger button presses. However, Albouy and colleagues (2008) investigated the implicit learning of a sequence of eye movements. In the implicit conditions, action sequences were often embedded in a larger set of movements so as to block explicit awareness of the task. Decreases in reaction time are indicative of learning during sequence blocks, relative to blocks where all button presses were random. Relatedly, a secondary task was also at times employed to further prevent participants from gaining explicit awareness of the sequence (Grafton et al., 2002). Under explicit learning conditions, participants were instructed that they would be learning a sequence and were aware of the task goals.

Visuomotor adaptation paradigms take two main forms. Most commonly, participants manipulate a hand-held joystick in order to move a small object to a target location. After a practice period, the visual feedback is rotated such that the feedback on the screen does not match the movements of the joystick (c.f. Anguera et al., 2007). Alternatively, participants may also be instructed to make pointing movements to a target while wearing prism distortion goggles (Luauté et al., 2009). In both cases, the visual feedback of movement is distorted.

In both verbal and spatial working memory tasks participants have to hold and manipulate information in mind over a span of a few seconds. Two of the most commonly used tasks are the n-back task and the Sternberg working memory task. The n-back task can be administered using either verbal or spatial stimuli (c.f. Thomas et al., 1999; Kim et al., 2003). In verbal tasks, letters (or numbers) are presented individually and subjects have to indicate whether the current letter matches what was presented “n” trials previously. In spatial tasks participants are asked to compare locations of stimuli across successive presentations. Also commonly used is the Sternberg working memory task (Sternberg, 1966). In this paradigm groups of letters are presented. After a delay period participants are presented with a letter and are asked to indicate whether or not that letter was part of the previously viewed set. Additionally, the included studies also employed tests of mental rotation (spatial working memory) as well as paced addition tasks (verbal working memory).

Importantly, across these task domains, participants were required to make their responses with the fingers and hand. The one exception was implicit sequence learning of eye movements (Albouy et al., 2008). In general, across domains the effectors used during the learning paradigms did not vary significantly. This is particularly important given the somatotopy within the cerebellum (Nitschke et al., 1996; Wiestler et al., 2011). Any differences in activation across these motor tasks cannot be attributed to differences in the effectors used in each task domain. With respect to working memory, all of the responses were made with the hands and fingers across tasks, although all of the studies included in our analyses also controlled for the motor responses.

### ALE meta-analysis

All analyses were completed using GingerALE 2.3 (www.brainmap.org/ale; Laird et al., 2005; Eickhoff et al., 2009). Foci within the cerebellum for each task type were combined into individual text files. Because all of the foci need to be in the same space, foci in Talairach space that were transformed using the Brett transform (mni2tal) were converted back to MNI space using the inverse of the Brett transform. Those that were transformed into Talairach space using the Lancaster transform (Lancaster et al., 2007; icbm2tal) were transformed back into MNI space, also using the inverse of this transform. Finally, in cases where there was no transform specified, or where data were initially normalized into Talairach space, the Lancaster icbm2tal transform was used to bring these foci into MNI space. Importantly, the icbm2tal is a newer transformation (Lancaster et al., 2007) and we were careful to ensure that this was used only on studies where it would have been initially available. Older work transformed with icbm2tal was restricted to studies that were initially normalized directly into Talairach space. These transformations were completed using the “convert foci” tool in GingerALE. Foci in MNI space within the cerebellum for each task type were combined into individual text files.

The text files were then entered into GingerALE. GingerALE automatically computes the ALE values for every voxel in the brain, and does so using an automatically determined full-width half-maximum (FWHM) value (Eickhoff et al., 2009). However, upon completion of the analyses, the FWHM value of each focus was reported to be between 9 and 10 mm. The ALE value was computed using permutation testing (5000 permutations) against the null-distribution of random spatial associations of foci across experiments (Eickhoff et al., 2009). The ALE scores resulting from this permutation testing are then used to assign *p*-values to the actual values of the input data. We used a false discovery rate of *p* < 0.05 to correct for multiple comparisons. Additionally, all clusters were set to a minimum of 50 mm^3^. We completed ALE analyses for visuomotor adaptation, explicit sequence learning, implicit sequence learning, spatial working memory, and verbal working memory. We completed additional ALE analyses on the subset of explicit motor sequence learning studies that looked at activation during the early and late stages of learning. Notably, because we were generally interested in the regions involved in motor learning, areas that showed decreases in activation over the course of learning were considered with those that showed increases in activation. While most studies specifically investigated increases in activation, there were a few investigations of decreases in activation, though there were not a sufficient number of foci to investigate these decreases separately.

GingerALE also allows for statistical comparisons between the ALE maps of two distinct sets of foci. We used this method to investigate areas of overlap between task domains. We were particularly interested in the conjunction analyses across different tasks. Specifically, we investigated potential regions of overlap between visuomotor adaptation and all studies of sequence learning (combining both explicit and implicit studies), visuomotor adaptation and spatial working memory, explicit sequence learning and verbal working memory, all sequence learning and verbal working memory, and the early and late phases of learning during explicit sequence learning. This was computed using 5000 permutations, and we again used a false discovery rate of *p* < 0.05 and minimum cluster size of 50 mm^3^.

The results were visualized using MRICron (http://www.mccauslandcenter.sc.edu/mricro/mricron/index.html) and overlaid on an MNI template brain. The peaks of the ALE clusters were localized using the (Schmahmann et al., 1999) atlas of the human cerebellum. Because we were combining studies using standard normalization procedures, we were unable to use the recently developed SUIT atlas (Diedrichsen, 2006; Diedrichsen et al., 2009). The implications of older cerebellar templates and standard normalization procedures are addressed further in the discussion.

## Results

### ALE peaks for motor learning and working memory tasks

**Figure 2:**
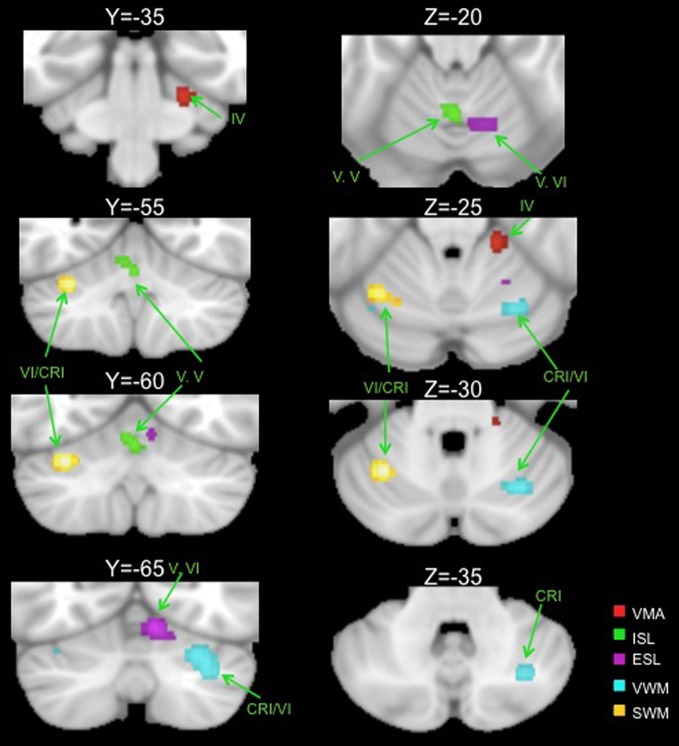
**Significant ALE clusters of activation for each examined task type are presented on coronal (left) and axial (right) slices of the cerebellum.** All clusters are thresholded and corrected for multiple comparisons using a false discovery rate *p* < 0.05. VMA, visuomotor adaptation; ISL, implicit sequence learning; ESL, explicit sequence learning; VWM, verbal working memory; SWM, spatial working memory; CRI, Crus I.

**Figure 3:**
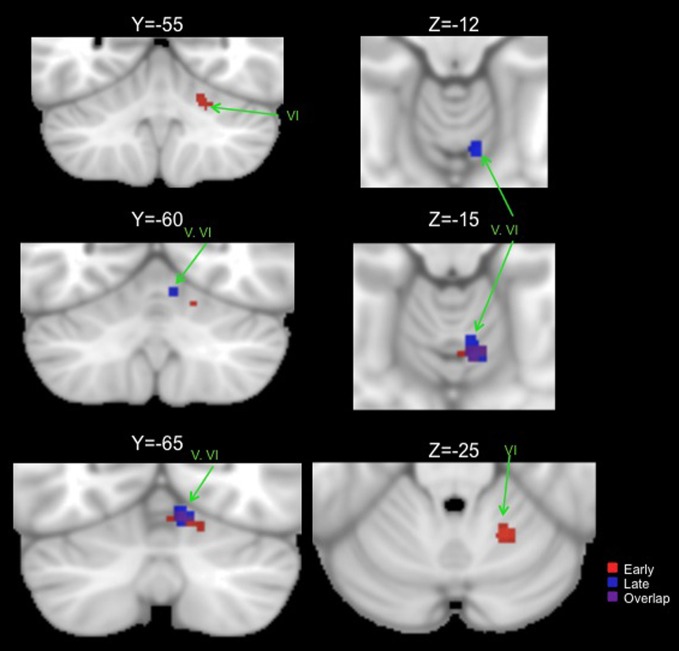
**Significant ALE clusters of activation for the early (red) and late (blue) phases of explicit sequence learning presented on coronal (left) and axial (right) slices of the cerebellum.** All clusters are thresholded and corrected for multiple comparisons using a false discovery rate *p* < 0.05.

Table 2 presents the peak coordinates, weighted centers, cluster sizes, and anatomical locations for the significant ALE maxima across each task domain. Figure 2 presents the ALE activation maps for visuomotor adaptation, explicit and implicit sequence learning, and spatial and verbal working memory. Figure 3 presents the ALE activation maps for early and late explicit sequence learning.

**Table 2:** **Peak ALE coordinates for each task category**.

**Cluster**	**Cluster size (mm^3^)**	**Extent and weighted center (*x*, *y*, *z*)**	**Local extrema (*x*, *y*, *z*)**	**Location**	**ALE value (× 10^−3^)**
**VISUOMOTOR ADAPTATION**					
Cluster 1	328	From (18, −40, −30) to (24, −32, 24) centered at (20.6, −36.01, −26.47)	(20, −36, −26)	Lobule IV	12.36
**IMPLICIT SEQUENCE LEARNING**					
Cluster 1	592	From (−8, −64, −24) to (4, −54, −16) centered at (−1.74, −58.34, −19.92)	(0, −60, −20)	Vermis lobule V	9.41
**EXPLICIT SEQUENCE LEARNING**					
Cluster 1	928	From (4, −70, −22) to (18, −60, −12) centered at (9.82, −64.95, −16.4)	(8, −66, −14)	Vermis/lobule VI	18.39
**EXPLICIT SEQUENCE LEARNING: EARLY LEARNING**					
Cluster 1	304	From (18, −56, −28) to (24, −50, −22) centered at (21.1, −53.47, −25.1)	(22, −54, −26)	Lobule VI	9.06
Cluster 2	216	From (4, −68, −20) to (16, −60, −14) centered at (11.22, −64.9, −17.09)	(8, −66, −16)	Vermis/lobule VI	7.72
**EXPLICIT SEQUENCE LEARNING: LATE LEARNING**					
Cluster 1	384	From (6, −70, −18) to (12, −60, −12) centered at (8.63, −64.93, −14.85)	(8, −66, −14)	Vermis/lobule VI	11.16
**VERBAL WORKING MEMORY**					
Cluster 1	1128	From (24, −68, −40) to (38, −62, −24) centered at (31.31, −65.39, −30.97)	(30, −66, −28)	Crus I/lobule VI border	37.3
**SPATIAL WORKING MEMORY**					
Cluster 1	704	From (−38, −64, −32) to (−24, −54, −24) centered at (−32.44, −58.75, −28.63)	(−34, −58, −28)	Lobule VI	19.62
			(−24, −64, −26)	Lobule VI	13.19

Though we were unable to investigate early vs. late learning activation in the visuomotor adaptation task, analysis of activations across the entire learning period resulted in one significant cluster in the anterior cerebellum, localized in lobule IV. Also located in the anterior lobe was a significant cluster associated with implicit sequence learning. However, this cluster was located along the midline in the vermis region of lobule V.

When combining across all studies and phases of learning, explicit sequence learning was associated with a large cluster in the vermis region of lobule VI, extending into lobule VI itself. During the early phase of explicit sequence learning there were two significant ALE clusters. One cluster was located more medially in the vermis region of lobule VI and extended laterally into lobule VI. The second cluster was located more laterally, and was inferior to the first cluster in lobule VI. During the late phase of explicit sequence learning, the activation was again more medial in the vermis of lobule VI and extending into lobule VI itself.

Finally, we investigated both spatial and verbal working memory. Spatial working memory processing activated a cluster in the left cerebellum in lobule VI, while verbal working memory processing activated a large cluster in the right cerebellum on the border between lobule VI and Crus I. These findings closely replicate those described by the meta-analysis performed by Stoodley and Schmahmann (2009), and are also consistent with the functional topography of the cerebellum that has been demonstrated using functional neuroimaging (Stoodley et al., 2010, 2012). Notably, there were no clusters in the inferior regions of the cerebellum as reported in recent meta-analyses (Stoodley and Schmahmann, 2009; E et al., in press). In part, this may be due to the number of additional studies included in our analysis [44 working memory studies, compared to the 8 and 26 used by Stoodley and Schmahmann (2009) and E et al. (in press), respectively]. Furthermore, this inferior region was associated most strongly with the Sternberg task (E et al., in press), as evidenced by comparisons across working memory tasks. Though many of the studies in our analyses employed variants of the Sternberg task, there were additional working memory tasks included, perhaps resulting in our null finding in the inferior cerebellum.

### Analysis of overlap across tasks

Conjunction analyses across sets of foci were carried out to investigate overlapping regions of the cerebellum across tasks. We investigated overlap between visuomotor adaptation and sequence learning (collapsing across all implicit and explicit studies), visuomotor adaptation and spatial working memory, all sequence learning and verbal working memory, explicit sequence learning and verbal working memory, and the early and late stages of explicit sequence learning. There was no significant overlap between any of these sets of foci with the exception of the early and late stages of explicit sequence learning. There was a significant cluster of overlap in the vermis region of lobule VI associated with both early and late explicit sequence learning (Table 3, Figure 3). However, there was no overlap between late learning and the more lateral lobule VI cluster associated with early explicit sequence learning.

**Table 3:** **Overlap of regions engaged during the early and late phases of explicit sequence learning**.

**Cluster**	**Cluster size (mm^3^)**	**Extent and weighted center (*x*, *y*, *z*)**	**Local extrema (*x*, *y*, *z*)**	**Location**	**ALE value (× 10^−3^)**
Cluster 1	112	From (6, −68, −18) to (12, −64, −14) centered at (8.86, −65.71, −15.71)	(8, −66, −16)	Vermis/Lobule VI	7.72

## Discussion

Using ALE meta-analysis, we investigated cerebellar involvement in multiple motor learning tasks, including visuomotor adaptation and both explicit and implicit motor sequence learning. We further investigated cerebellar regions associated with working memory processes and their potential involvement in motor learning. Our results provide evidence consistent with the role of the anterior cerebellum in motor tasks, though our findings did not indicate overlapping engagement of cerebellar regions for both working memory processes and motor learning. The anterior cerebellum, particularly along the midline, was active across studies of explicit and implicit sequence learning, with an additional anterior region associated with visuomotor adaptation. The distinct regions associated with these motor tasks provide conceptual support for the MOSAIC theory (Wolpert and Ghahramani, 2000; Imamizu et al., 2003) of modular internal models in the cerebellum. Additionally, we provide support for the involvement of more lateral and posterior regions of the cerebellum in explicit sequence learning. This is consistent with prior work indicating an additional homunculus in this region associated with the performance of complex motor tasks (Schlerf et al., 2010). However, notably, we found no overlap between regions associated with spatial and verbal working memory processes and any of the motor learning tasks we investigated, despite our previous work demonstrating correlations between an individual's working memory capacity and their motor learning of these tasks (Bo and Seidler, 2009; Bo et al., 2009, 2011, 2012; Anguera et al., 2010, 2011).

### The cerebellum and internal models of action

It has been proposed that the cerebellum is important for the formation of internal models of actions (Miall et al., 1993; Miall and Wolpert, 1996; for reviews see Ramnani, 2006; Ito, 2008). According to the MOSAIC theory, these internal models are modularly represented in the cerebellum for motor actions as well as cognitive processes (Wolpert and Ghahramani, 2000; Imamizu et al., 2003). Supporting this theory, we found that cerebellar activation was distinct for each of multiple motor learning task types.

With that said, it is important to note that in both implicit sequence learning and visuomotor adaptation, we were unable to subdivide the collected foci into the early and late phases of learning. We were therefore unable to investigate differences in the activated regions that would be indicative of the formation of new internal models of the learned skills. One alternative possibility is that the different regions of activation across tasks were due to the motor somatotopy within the anterior cerebellum (Nitschke et al., 1996; Buckner et al., 2011; Wiestler et al., 2011). There is a general body representation within this region; even individual finger representations can be discerned (Wiestler et al., 2011). The distinct regions may be due to the overall motor demands of the learning tasks, and variability may be associated with different effector usage for task performance. For example, sequence learning tasks typically involved tapping with individual fingers, whereas visuomotor adaptation often required the manipulation of a joystick with either several fingers or the whole hand. As such, distinct anterior cerebellar regions may have been engaged.

Lastly with respect to the localization of these activations, across these motor tasks activity across studies was generally confined to anterior regions of the cerebellum. This is consistent with the functional topography of the cerebellum wherein motor representations are located in the anterior cerebellum, as well as in lobules VIIIa and VIIIb in the posterior cerebellum (Schmahmann and Sherman, 1998; Gerwig et al., 2003; Stoodley and Schmahmann, 2009; Stoodley et al., 2010, 2012). Though we did not see any activation clusters in the secondary, more posterior motor representation, it has recently been suggested that the function of the posterior region is different than that of the anterior motor representation, and it may be less important for motor control (Donchin et al., 2012). Additionally, our midline clusters associated with both implicit and explicit sequence learning are in a cerebellar region where gray matter volume has been linked to the degree to which individuals learn a new motor sequence (Steele et al., 2012).

### Working memory and motor learning in the cerebellum

As the early stage of learning is thought to rely on cognitive processes (Fitts and Posner, 1967; Anderson, 1982), we predicted that there would be overlap between areas engaged in spatial and verbal working memory and those associated with motor learning. However, this was not supported by the results. This is somewhat surprising given the relationship between working memory capacity and both sequence learning and visuomotor adaptation (Bo and Seidler, 2009; Bo et al., 2009, 2011, 2012; Anguera et al., 2010), and the recruitment of neural resources associated with working memory during the early phase of visuomotor adaptation (Anguera et al., 2010).

Lateral and posterior cerebellar regions are thought to communicate with the prefrontal cortex through closed loop circuits (Ramnani, 2006). These regions are also implicated in both spatial and verbal working memory tasks as demonstrated in our analyses, consistent with prior work (Chen and Desmond, 2005a,b; Stoodley and Schmahmann, 2009; Stoodley et al., 2010, 2012). One may then imagine that if working memory circuits are engaged during the early phases of motor learning, the cerebellar components of those circuits may also be brought online. In fact, in learning novel skills that may require more cognitive resources, new internal models are formed, but they seem to be in more lateral regions of the cerebellum (Imamizu et al., 2000, 2003). Perhaps, because we were unable to differentiate between the early and late learning phases in the visuomotor adaptation task and in implicit sequence learning, we were unable to differentiate regions that may be associated with more general motor execution from those associated with the formation of a new internal model. Similarly, in our analyses we treated regions that showed decreases in activation over the course of learning in the same way as those that showed parametric increases in activation over the course of learning. A greater number of foci in each category would allow for differentiation and may indicate that areas of decrease are associated with the cognitive demands of early learning (Anguera et al., 2010), while those that exhibit increases may be more associated with the formation of new internal models.

We were able to investigate the early and late phases of explicit sequence learning. During the early phase of learning there were two significant cerebellar clusters, one of which was more lateral and inferior in lobule VI. Though there was no overlap with regions associated with either spatial or verbal working memory, this region is consistent with an area reported to show increased activation during the performance of more complex motor tasks (Schlerf et al., 2010). In this investigation during the complex task participants executed sequences of finger flexion and extension. This was compared to a simple task requiring the simultaneous flexion and extension of multiple digits at once. In our data, as with those of Schlerf and colleagues (2010), activity was localized in lobule VI. Lobule VI has been implicated in working memory task performance (Chen and Desmond, 2005a,b), and the resting state networks of this region include correlations with both pre-motor and lateral prefrontal cortical regions (Bernard et al., 2012). Thus, though the activation in lobule VI associated with early explicit sequence learning does not directly overlap with those associated with verbal or spatial working memory, lobule VI does seem to be involved in higher cognitive processing. However, given that we averaged across multiple studies and foci, there may be some overlap on an individual study level. Our cluster in this region associated with early explicit sequence learning may therefore reflect some of the cognitive demands associated with this stage of motor skill learning. Finally, the more superior and medial early learning cluster overlapped with that of late learning. This may be more indicative of a newly formed internal model.

### Limitations

The use of meta-analysis to investigate activations across studies has some limitations. First is that of study selection. While we defined our study selection criteria based on age and study parameters to eliminate any potential bias, there may be additional available studies that merit inclusion but were not found based on our search terms. Our results are limited to those studies that are available in Pubmed within our given search parameters. Furthermore, a variety of different tasks have been used to investigate working memory and motor learning. For example, verbal working memory may be measured using an n-back task, a Sternberg task, or the paced auditory serial addition task. Because we were interested in the general processes, and not the specific tasks themselves we collapsed across these task types. Notably, there was less variability across sequence learning tasks and visuomotor adaptation paradigms, but this may still impact our results.

Second, combining multiple studies means that data are often normalized to different brain templates, or normalized and transformed from one template to another. Though algorithms are available to bring data across several studies into the same anatomical space, perfect registration across subjects cannot be guaranteed. Relatedly, the acquisition and other processing parameters vary across these studies. Indeed, because we included both PET and fMRI results in our analysis, this is particularly pertinent. Importantly however, the ALE algorithm employed here includes random-effects modeling designed to account at least in part for these limitations (Eickhoff et al., 2009).

Lastly, it is worth noting that the studies included in this meta-analysis relied primarily on standard affine transformations for normalization. These methods implemented in common neuroimaging packages often result in poor alignment between cerebellar regions (Diedrichsen, 2006). Recently, Diedrichsen and colleagues have created a spatially-unbiased atlas and updated normalization procedure to improve cerebellar registration (Diedrichsen, 2006; Diedrichsen et al., 2009). Because of the relative novelty of this normalization procedure and the span of time over which our studies originate, use of this procedure was rare in the studies we sampled. Most of the investigations we included used more standard normalization parameters and templates. Thus, our results should be interpreted with some caution as the actual locations may vary slightly due to normalization procedures.

## Conclusions

Here, we investigated the role of the cerebellum in motor skill learning using ALE meta-analysis. We combined foci across studies investigating visuomotor adaptation, motor sequence learning (explicit and implicit), and verbal and spatial working memory. We demonstrated that distinct motor tasks engaged differing regions of the cerebellum, providing further evidence for the notion that the internal models of the cerebellum are formed in a modular manner. Furthermore, these regions were generally limited to the anterior portion of the cerebellum, consistent with its general functional topography. Additionally, we also found that although the cerebellum seems to engage regions associated with the lateral prefrontal cortex and working memory performance during the early stage of explicit sequence learning, this region did not overlap with any of the significant ALE clusters associated with the working memory domains (verbal and spatial) that we investigated here. In general, this provides support for the role of the cerebellum in processing the cognitive demands of the early phases of sequence learning, but further investigations are needed to see if this generalizes to other domains of motor skill learning. In particular, more fine-grained studies investigating cerebellar functional modularity across tasks and their associated timecourses are warranted.

### Conflict of interest statement

The authors declare that the research was conducted in the absence of any commercial or financial relationships that could be construed as a potential conflict of interest.
